# Introducing robotic surgery in a low-volume, community hospital: outcomes of the first 50 ventral hernia repairs (TARUP)

**DOI:** 10.3389/jaws.2026.15942

**Published:** 2026-06-04

**Authors:** Jult Anton, Maertens Vicky, Melsens Elodie, Verrelst Lynn, Van Kerschaver Olivier, Fierens Kjell

**Affiliations:** 1 General Surgery, Ghent University Hospital, Ghent, Belgium; 2 vzw AZ Sint-Lucas & Volkskliniek, Ghent, Belgium; 3 General Surgery, Universiteit Gent, Ghent, Belgium

**Keywords:** learning curve, robotic, robotic abdominal wall repair, TARUP, ventral hernia repair

## Abstract

**Background:**

Robotic transabdominal retromuscular umbilical prosthetic repair (TARUP) is increasingly used for minimally invasive retromuscular ventral hernia repair, but data on implementation and learning curves in low-volume, non-tertiary community hospitals remain limited. We report early outcomes and operative efficiency during program initiation.

**Methods:**

Retrospective single-center cohort study in a non-specialist, non-tertiary community hospital. The first 50 consecutive robotic TARUP procedures (2022–2024) were screened; 43 patients were included after predefined exclusions. All hernias were primary midline ventral hernias (EHS width class W1). The primary outcome was operative time (skin-to-skin). Secondary outcomes included non-operative OR time, total OR time, mesh area, early postoperative pain (VAS at 2 h), length of stay (LOS), same-day discharge, and 30-day complications. Learning curves were assessed using chronological tertiles and CUSUM analysis.

**Results:**

Retrospective single-center cohort study in a non-specialist, non-tertiary community hospital. The first 50 consecutive robotic TARUP procedures (2022–2024) were screened; 43 patients were included after predefined exclusions. All hernias were primary midline ventral hernias (EHS width class W1). The primary outcome was operative time (skin-to-skin). Secondary outcomes included non-operative OR time, total OR time, mesh area, early postoperative pain (VAS at 2 h), length of stay (LOS), same-day discharge, and 30-day complications (Clavien–Dindo). Learning curves were assessed using chronological tertiles and CUSUM analysis.

**Conclusion:**

In a low-volume, non-tertiary community hospital, robotic TARUP showed a clear learning curve with improved operative efficiency over chronological experience and declining minor complications, alongside favorable 30-day recovery outcomes in selected smaller, primary midline ventral hernias. Longer follow-up and broader hernia complexity are needed to assess durability and generalizability.

## Introduction

Robotic-assisted surgery has rapidly expanded in abdominal wall reconstruction, combining the principles of minimally invasive surgery with the technical precision of open retromuscular techniques. The introduction of robotic platforms allows for improved dexterity, three-dimensional visualization, and enhanced ergonomics, which may be particularly advantageous in complex abdominal wall repair [1].

Within this context, the transabdominal retromuscular approach with prosthetic reinforcement (TARUP) has emerged as a widely adopted technique for robotic application. As a minimally invasive adaptation of the Rives–Stoppa repair, robotic TARUP enables wide retrorectus mesh overlap while avoiding intraperitoneal mesh placement [2, 3]. Observational series and comparative studies have reported favorable short-term outcomes after robotic retromuscular repair, including low wound morbidity and short hospital stay, although operative efficiency and resource utilization remain important considerations [4–6].

Despite these advantages, robotic ventral hernia repair remains a double-edged sword. Longer operative times and higher procedural costs have raised concerns about cost-effectiveness and broad accessibility [7]. Moreover, the technique is associated with a distinct learning curve, as demonstrated by cumulative sum (CUSUM) and observational analyses, which show operative efficiency improving markedly after the first 20–30 cases [2, 8]. Importantly, this learning curve does not only apply to the surgeon but also involves the anesthesiology, nursing, and perioperative teams [9].

The clinical impact of implementing robotic abdominal wall reconstruction in non-academic or low-volume community hospital settings has only recently been explored. Initial reports suggest that short-term outcomes remain favorable during the early learning phase, provided that structured training and careful patient selection are ensured [9, 10]. However, evidence on the introduction of robotic abdominal wall reconstruction outside high-volume academic centers remains limited. In particular, data describing early outcomes during program initiation in community hospitals are scarce. Against this background, our institution introduced robotic TARUP as the index procedure following acquisition of a robotic platform. The present study reports the early 30-day outcomes of the first consecutive robotic TARUP cases and evaluates the associated learning curve using chronological tertiles and CUSUM analysis, focusing on operative time, length of stay, early postoperative pain, mesh characteristics, and 30-day complications.

## Methods

### Study design

This study was designed as a retrospective, single-center cohort study evaluating early outcomes and the learning curve following implementation of robotic transabdominal retromuscular umbilical prosthetic repair (TARUP) for elective midline ventral hernia repair in a non-specialist, community hospital. The cohort consisted of the first 50 consecutive robotic TARUP procedures performed after program launch (2022–2024). After predefined exclusions, 43 patients were included for analysis. No external control group or case-matched comparator cohort was included; all analyses were performed within the study cohort based on chronological case order.

The primary outcome was operative time (skin-to-skin). Secondary outcomes included length of stay (LOS), same-day discharge rate, early postoperative pain (visual analogue scale, VAS, at 2 h), mesh area, non-operative operating room time, and 30-day postoperative complications.

The learning curve was assessed using chronological case order, comparing outcomes across tertiles and visualizing operative time trends using a cumulative sum (CUSUM) chart.

### Setting and surgical team

All procedures were performed in a non-academic community hospital after acquisition of a da Vinci Xi® robotic platform (Intuitive Surgical, Sunnyvale, CA, USA). In the Belgian healthcare context, the term “community hospital” is used here to denote a non-university, general acute-care hospital. Robotic TARUP was selected as the index procedure for initiation of the hospital’s robotic program because it allows retromuscular mesh placement through a minimally invasive approach and offers a standardized workflow suitable for stepwise implementation.

The program was introduced in a structured manner. Two attending general surgeons with extensive experience in advanced laparoscopy but no prior clinical experience with robotic abdominal wall reconstruction performed the procedures. Before the first clinical case, both surgeons completed a formal training pathway for the robotic platform, including console simulation, dry-lab exercises, and hands-on courses. Initial cases were performed with a consistent OR setup and standardized port placement and docking strategy. A dedicated scrub nurse and circulating nurse team was progressively familiarized with robotic-specific workflows, including patient positioning, docking, instrument exchanges, and robotic draping.

Anesthesiology care and perioperative nursing were delivered by teams without prior routine exposure to robotic abdominal wall reconstruction at program start. Non-operative operating room time was therefore recorded to reflect team- and workflow-related components of early implementation (including anesthetic induction, positioning, robotic docking, and transfer). Throughout the study period, the same institutional perioperative pathways were used for elective ventral hernia surgery, including standardized postoperative monitoring and discharge criteria.

The study was approved by the ethics committee of AZ Sint-Lucas Ghent with trial number 2025-26.

### Participants

Consecutive adult patients (≥18 years) undergoing elective robotic ventral hernia repair during the implementation period (2022–2024) were screened for eligibility. Patients were included in the analytic cohort if they underwent robotic TARUP for a primary midline ventral hernia with hernia characteristics classified according to the European Hernia Society (EHS) classification, including midline location (M1–M5) and defect width category (W1 <4 cm, W2 4–10 cm, W3 >10 cm). Recurrent hernias were not included in this early implementation cohort.

Among the first 50 consecutive robotic ventral hernia procedures performed during the study period, seven were excluded from the final analytic cohort according to predefined criteria: four involved non-midline hernias, two did not require retromuscular mesh reinforcement consistent with the TARUP concept (mesh area <100 cm^2^), and one was performed by an operator who contributed only a single case during the implementation phase. The final analytic cohort therefore comprised 43 patients.

All patients were scheduled for routine postoperative follow-up in the outpatient clinic approximately 4 weeks after surgery. Early outcomes were assessed up to 30 days postoperatively.

### Surgical technique

Robotic TARUP was performed using a standardized lateral transabdominal retromuscular approach. The robotic platform (da Vinci Xi®, Intuitive Surgical, Sunnyvale, CA, USA) was docked from the patient’s left side in all cases. Patients were positioned supine with both arms tucked under general anesthesia. Pneumoperitoneum was maintained at 12 mmHg for exposure and temporarily reduced to 8 mmHg during midline reconstruction to facilitate tension-free closure.

Three 8-mm robotic trocars were placed laterally on the right abdominal wall in a standardized configuration; no assistant port was used. Access to the retromuscular plane was obtained via a lateral incision through the peritoneum and posterior rectus sheath on the right side. The right retrorectus space was developed cranio-caudally by blunt and sharp dissection, with reduction of hernia contents and adhesiolysis as required.

A crossover to the contralateral retrorectus space was performed, creating a bilateral retromuscular pocket suitable for midline reconstruction and mesh placement.

Hernia defect width was assessed intraoperatively and documented. The linea alba was reconstructed using a running barbed suture (Stratafix™ 1, Ethicon), performed under reduced pneumoperitoneum (8 mmHg). Midline plication was systematically performed in all cases, including patients without a clinically relevant rectus diastasis.

A self-fixating mesh (Progrip™, Medtronic) was introduced and positioned in the retromuscular plane aiming for broad overlap in all directions. No additional mesh fixation (sutures or tacks) was used. The posterior layer/peritoneum was closed with a running barbed suture (V-Loc™ 2-0, Medtronic). Any peritoneal defects encountered were repaired to maintain complete separation of the mesh from the intraperitoneal compartment. No drains were placed.

### Variables and data collection

Data were extracted retrospectively from the electronic surgical records, anesthesia charts, and nursing documentation and entered into a dedicated study database. Two investigators independently verified key variables (operative time, length of stay, mesh characteristics, pain score, and complications) against the source records.

Collected baseline variables included age, sex, body mass index (BMI), and ASA physical status classification. Hernia characteristics were documented using the European Hernia Society (EHS) classification, including midline location (M1–M5) and defect width category (W1–W3). Hernia defect width was recorded based on intraoperative assessment. Presence of rectus diastasis was recorded when available from preoperative imaging and/or intraoperative assessment. Detailed comorbidity variables (e.g., hypertension, diabetes, antiplatelet/anticoagulant therapy, or immunosuppression) were not consistently documented in a standardized manner across the early implementation period and were therefore not included in the analysis.

Operative time was defined as skin-to-skin time (incision to completion of skin closure) and recorded in minutes. Mesh size was recorded as mesh area (cm^2^) based on the implanted mesh dimensions. Non-operative operating room (OR) time was defined as the time components not included in skin-to-skin operative time and comprised anesthetic induction, patient positioning, robotic docking/undocking, extubation, and transfer. Length of stay (LOS) was recorded in days. Same-day discharge was defined as discharge on the calendar day of surgery. Early postoperative pain was assessed using a 0–10 visual analogue scale (VAS) at 2 h postoperatively, as documented in the standardized nursing chart. Postoperative complications were recorded up to 30 days after surgery and defined as any deviation from the expected postoperative course. Complications were categorized clinically (e.g., seroma, hematoma, surgical site infection) and graded according to the Clavien–Dindo classification when applicable. Follow-up was based on routine outpatient assessment approximately 4 weeks postoperatively combined with review of readmissions and emergency department visits within the 30-day postoperative period.

### Statistical analysis

Continuous data are presented as median (interquartile range, IQR) and categorical data as number (percentage). Normality was assessed using the Shapiro–Wilk test.

For learning-curve assessment, cases were divided into chronological tertiles based on the order of procedures. Differences across tertiles were evaluated using the Kruskal–Wallis test for continuous variables and the Fisher’s exact test (or Chi-square test when appropriate) for categorical variables. Post-hoc pairwise comparisons were performed when relevant.

To provide an overall visualization of operative performance over time, a cumulative sum (CUSUM) chart was constructed for operative time. The target value was defined as the mean operative time of the entire cohort. For each case, the deviation from the target (operative time–target) was calculated and cumulatively summed across consecutive procedures; upward trends indicate operative times above the target and downward trends indicate operative times below the target. CUSUM was used as a descriptive learning-curve visualization rather than a formal inferential model.

All tests were two-sided and a p-value <0.05 was considered statistically significant. Analyses were performed using SPSS v28 (IBM Corp., Armonk, NY, USA).

## Results

### Patient characteristics

A total of 43 patients undergoing elective robotic TARUP were included, all presenting with primary midline ventral hernias. Median age was 49.0 years (IQR 38.5–61.0) and median BMI was 29.8 kg/m^2^ (IQR 25.4–34.0). The cohort included 22 men (51.2%) and 21 women (48.8%). All patients were classified as ASA I–II (ASA I: 19/43, 44.2%; ASA II: 24/43, 55.8%). According to the European Hernia Society (EHS) classification, hernia location was exclusively midline M2–M3 (M2: 17/43, 39.5%; M3: 25/43, 58.1%; M4: 1/43, 2.3%). All defects were categorized as W1 (<4 cm), and all hernias treated were primary Rectus diastasis was present in 23/43 (53.5%) (Table 1).

**TABLE 1:** Baseline characteristics.

Parameter		Value
Median age, years		49.0 (38.5–61.0)
Sex (%)	Male	22 (51.8%)
	Female	21 (48.2%)
Median BMI (kg/m2)		29.8 (25.4–34.0)
ASA	1	19 (44.2%)
	2	24 (55.8%)
EHS classficiation	pM2W1	17 (39.5%)
	pM3W1	25 (58.1%)
	pM4W1	1 (2.3%)
Rectus diastasis	Yes	23 (53.5%)
	No	20 (46.5%)

Values are median (IQR) or n (%). Same-day discharge defined as discharge on the day of surgery.

### Perioperative outcomes

Median operative time (skin-to-skin) was 141.0 min (IQR 122.5–170.5). Median non-operative operating room time was 36.0 min (IQR 29.0–45.5), resulting in a median total OR time of 178.0 min (IQR 161.5–201.0). Median implanted mesh area was 210.0 cm^2^ (IQR 174.5–255.0). Early postoperative pain was low, with a median VAS score at 2 h of 2.0 (IQR 2.0–3.0). Median length of stay was 0.53 days (IQR 0.45–1.22), and 24/43 (55.8%) patients were discharged the same day (Table 2).

**TABLE 2:** Perioperative outcomes.

Outcome	Value
Operative time (skin-to-skin), min	141.0 (122.5–170.5)
Non-operative OR time, min	36.0 (29.0–45.5)
Total OR time, min	178.0 (161.5–201.0)
Mesh area, cm^2^	210.0 (174.5–255.0)
VAS pain score at 2 h (0–10)	2.0 (2.0–3.0)
Length of stay, days	0.53 (0.45–1.22)
Same-day discharge	24 (55.8%)

Values are median (IQR) or n (%). Same-day discharge defined as discharge on the day of surgery.

### Learning curve analysis

For learning curve assessment, procedures were divided into chronological tertiles based on case order (T1 n = 15; T2 n = 14; T3 n = 14). Median operative time differed across tertiles (184.0 [151.5–219.0] min in T1, 122.5 [108.0–133.0] min in T2, and 142.5 [132.5–149.5] min in T3; p = 0.001). Median total OR time also differed across tertiles (198.0 [179.0–251.0] min, 165.0 [149.2–175.5] min, and 181.0 [165.8–188.0] min, respectively; p = 0.015). Non-operative OR time, mesh area, VAS at 2 h, and length of stay did not differ significantly between tertiles (Table 3).

**TABLE 3:** Values are median (IQR) or n/N (%).

Outcome	T1 (n = 15)	T2 (n = 14)	T3 (n = 14)	p-value
Operative time (skin-to-skin), min	184.0 (151.5–219.0)	122.5 (108.0–133.0)	142.5 (132.5–149.5)	0.001
Non-operative OR time, min	30.0 (20.0–45.5)	36.5 (26.8–53.5)	36.0 (31.5–43.2)	0.585
Total OR time, min	198.0 (179.0–251.0)	165.0 (149.2–175.5)	181.0 (165.8–188.0)	0.015
Mesh area, cm^2^	225.0 (172.5–300.0)	207.0 (153.0–247.5)	202.5 (180.5–232.5)	0.723
VAS pain score at 2 h (0–10)	3.0 (2.0–3.5)	2.5 (2.0–3.0)	2.0 (2.0–4.0)	0.853
Length of stay, days	1.18 (0.45–1.38)	0.51 (0.46–0.97)	0.54 (0.45–1.18)	0.468
Same-day discharge	6/15 (40.0%)	10/14 (71.4%)	8/14 (57.1%)	0.233
Any 30-day complication	7/15 (46.7%)	0/14 (0.0%)	2/14 (14.3%)	0.006
Operating surgeon: 1, n (%)	7/15 (46.7%)	6/14 (42.9%)	8/14 (57.1%)	-

Continuous variables: Kruskal–Wallis test. Categorical variables: χ^2^ test. No p-value shown for descriptive distribution rows.Values are median (IQR) or n/N (%). Continuous variables: Kruskal–Wallis test. Categorical variables: χ2 test. No p-value shown for descriptive distribution row.

Same-day discharge occurred in 6/15 (40.0%) patients in T1, 10/14 (71.4%) in T2, and 8/14 (57.1%) in T3 (p = 0.233). Any 30-day complication occurred in 7/15 (46.7%) patients in T1, 0/14 (0.0%) in T2, and 2/14 (14.3%) in T3 (p = 0.006). Operating surgeon distribution across tertiles is shown in Table 3.

**FIGURE 1:**
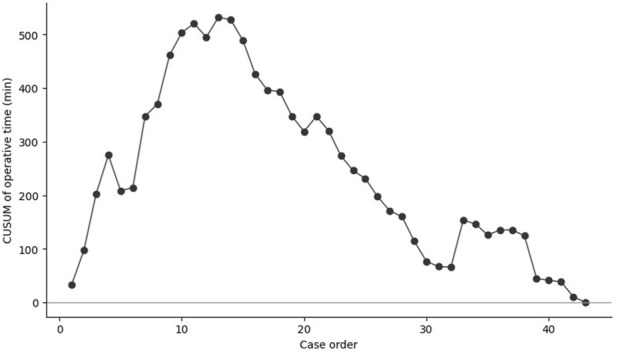
CUSUM plot of operative time. Cumulative sum (CUSUM) chart displaying operative time (skin-to-skin) in chronological case order. The target value was defined as the mean operative time of the entire cohort; each point represents the cumulative sum of deviations from this target across consecutive procedures. An upward slope indicates operative times above the target, whereas a downward slope indicates operative times below the target.

A CUSUM chart of operative time is shown in Figure 1. The cumulative sum increased during the early cases (maximum at case 13) and decreased thereafter.

### Thirty-day postoperative complications

Within 30 days, 34/43 (79.1%) patients had no postoperative complications. Overall, 9/43 (20.9%) patients experienced at least one complication. The most frequently recorded events were seroma (4/43, 9.3%) and hematoma (4/43, 9.3%). A surgical site infection occurred in 1/43 (2.3%). All recorded complications were graded as Clavien–Dindo I–II. Complications are summarized in Table 4.

**TABLE 4:** Thirty-day postoperative complications.

Complications	n (%)
Any	9 (20.9%)
Seroma	4 (9.3%)
Hematoma	4 (9.3%)
Surgical site infection	1 (2.3%)
No complication	34 (79.1%)

Values are n (%). Complications were recorded up to 30 days postoperatively and graded according to Clavien–Dindo (all grade I–II).

## Discussion

This retrospective single-center cohort study describes the introduction of robotic TARUP in a non-specialist, non-tertiary community hospital, with the primary aim of characterizing the learning curve during early implementation. Using both chronological tertiles and CUSUM visualization, we observed a clear improvement in operative efficiency over case order, while maintaining acceptable 30-day outcomes in a selected cohort of primary midline ventral hernias, all EHS W1.

The principal finding of this study is the measurable learning effect after introduction of robotic TARUP outside a high-volume tertiary environment. Operative time differed significantly across chronological tertiles and the CUSUM curve demonstrated an early accumulation followed by a turning point in the early phase and subsequent downward trend, consistent with increasing efficiency as case experience accrued. In the context of TARUP, Muysoms et al. previously described operative time dynamics during the learning curve, supporting operative time as a meaningful metric to capture early proficiency gains [2]. Our findings extend this concept to a non-tertiary, community hospital environment and underscore that learning effects are demonstrable even in lower-volume settings, provided technique and workflow are standardized.

An important implementation-specific observation is that non-operative OR time did not show a statistically significant change across tertiles. Non-operative time reflects team- and system-dependent steps (induction, positioning, docking/undocking, transfer). This distinction is particularly relevant in community hospitals, where robotic abdominal wall reconstruction is often introduced *de novo* at the level of the entire perioperative team. Similar program initiation experiences in non-academic settings highlight the role of structured training pathways and repeated team exposure in optimizing workflow [9]. Even in specialized hernia centers, introduction of a robotic program has measurable clinical impact and requires system-level adaptation beyond surgeon learning alone [10]. Together, these observations support the concept that the “learning curve” in early implementation is multi-layered: operative efficiency improves, while workflow optimization may require additional time and dedicated team standardization.

Beyond operative efficiency, we observed a decrease in 30-day complications across tertiles, with most events occurring early in the series. All recorded complications were minor (Clavien–Dindo I–II), with seroma and hematoma being the most common and a low rate of surgical site infection. A reduction in postoperative deviations over chronological experience is consistent with the concept that early implementation involves refinement of patient selection, tissue handling, hemostasis, and perioperative pathways. Reported short-term morbidity profiles in robotic ventral hernia repair are generally acceptable in selected populations, and minor wound-related events are commonly described in this field [11, 12]. Given that our cohort comprised exclusively W1 defects, these findings should be interpreted in the context of limited hernia complexity.

The retrospective single-center design and modest sample size limit generalizability and statistical power. The cohort comprised exclusively EHS W1 defects, limiting applicability to larger or more complex ventral hernias. Comorbidity variables beyond ASA were not consistently captured in a standardized manner, limiting risk stratification. Follow-up was limited to 30 days and therefore does not address recurrence, bulging, chronic pain, or longer-term patient-reported functional outcomes. Strengths include consecutive case inclusion during the initiation phase, a standardized technique, clear separation of baseline characteristics from perioperative outcomes, and complementary learning curve methods (tertiles and CUSUM) to describe performance evolution [1, 2]. Importantly, reporting from a non-tertiary community hospital provides a perspective that is underrepresented in the current TARUP literature.

## Conclusion

In a low-volume, non-tertiary community hospital, robotic TARUP for smaller, primary midline ventral hernias demonstrated a clear learning curve, with improved operative efficiency over chronological experience as shown by tertile comparisons and CUSUM visualization. Short-term recovery was favorable, with low early pain scores and frequent same-day discharge, and 30-day morbidity remained limited to minor (Clavien–Dindo I–II) events. Complication rates decreased across tertiles during the implementation phase. These findings support the feasibility of introducing robotic TARUP in a community hospital environment using a standardized technique and structured perioperative workflow. Further multicenter studies including broader hernia complexity and longer follow-up, incorporating recurrence and patient-reported outcomes, are required to evaluate durability and generalizability of these early implementation results.

## Data Availability

The raw data supporting the conclusions of this article will be made available by the authors, without undue reservation.
